# Transcriptome-Wide Identification and Response Pattern Analysis of the *Salix integra* NAC Transcription Factor in Response to Pb Stress

**DOI:** 10.3390/ijms241411334

**Published:** 2023-07-12

**Authors:** Yue Xin, Ruifang Huang, Meng Xu, Li’an Xu

**Affiliations:** 1 Co-Innovation Center for Sustainable Forestry in Southern China, Key Laboratory of Forest Genetics and Biotechnology Ministry of Education, Nanjing Forestry University, Nanjing 210037, China; xiny@njfu.edu.cn (Y.X.); xum@njfu.edu.cn (M.X.); 2 Willow Nursery of the Jiangsu Provincial Platform for Conservation and Utilization of Agricultural Germplasm, Jiangsu Academy of Forestry, Nanjing 211153, China; aion126@126.com

**Keywords:** NAC, transcription factor, *Salix integra*, Pb stress, stress response pattern

## Abstract

The NAC (NAM-ATAF1/2-CUC) transcription factor family is one of the largest plant-specific transcription factor families, playing an important role in plant growth and development and abiotic stress response. As a short-rotation woody plant, *Salix integra* (*S. integra*) has high lead (Pb) phytoremediation potential. To understand the role of NAC in *S. integra* Pb tolerance, 53 *SiNAC* transcripts were identified using third-generation and next-generation transcriptomic data from *S. integra* exposed to Pb stress, and a phylogenetic analysis revealed 11 subfamilies. A sequence alignment showed that multiple subfamilies represented by TIP and ATAF had a gene that produced more than one transcript under Pb stress, and different transcripts had different responses to Pb. By analyzing the expression profiles of *SiNACs* at 9 Pb stress time points, 41 of 53 *SiNACs* were found to be significantly responsive to Pb. Short time-series expression miner (STEM) analysis revealed that 41 *SiNACs* had two significant Pb positive response patterns (early and late), both containing 10 *SiNACs*. The *SiNACs* with the most significant Pb response were mainly from the ATAF and NAP subfamilies. Therefore, 4 and 3 *SiNACs* from the ATAF and NAP subfamilies, respectively, were selected as candidate Pb-responsive *SiNACs* for further structural and functional analysis. The RT-qPCR results of 7 transcripts also confirmed the different Pb response patterns of the ATAF and NAP subfamilies. SiNAC004 and SiNAC120, which were randomly selected from two subfamilies, were confirmed to be nuclear localization proteins by subcellular localization experiments. Functional prediction analysis of the associated transcripts of seven candidate *SiNACs* showed that the target pathways of ATAF subfamily SiNACs were “sulfur metabolism” and “glutathione metabolism”, and the target pathways of NAP subfamily SiNACs were “ribosome” and “phenylpropanoid biosynthesis”. This study not only identified two NAC subfamilies with different Pb response patterns but also identified Pb-responsive *SiNACs* that could provide a basis for subsequent gene function verification.

## 1. Introduction

NAC (NAM-ATAF1/2-CUC) is one of the largest transcription factor families, and it exists specifically in plants. The typical structure of the NAC protein is a highly conserved NAC domain containing approximately 150 amino acids at its N-terminus and a highly variable transcriptional regulatory region at its C-terminus [1]. The NAC domain is composed of five subdomains: A, B, C, D, and E. Subdomains A, C, and D are more conserved and may be involved in dimer formation and DNA binding. However, there were more variations in subdomains B and E, which may be related to the functional diversity of the NAC protein [2,3].

Since the first NAC transcription factor, NAM (related to meristem and primordium location), was discovered in tomato [4], researchers have found that the NAC protein is not only related to lateral root development [5,6], flowering [7], leaf senescence [8,9], and other growth and development processes of plants but also plays an important role in plant response to abiotic stresses such as drought [10,11], high salt [12], and ABA [13]. In recent years, some studies have also shown that NAC plays a role in plant tolerance to heavy metals. For example, *ANAC004* [14] and *ANAC017* [15] regulate cadmium and aluminium tolerance, respectively, in *Arabidopsis thaliana* (*A. thaliana*); *OsNAC300* can improve cadmium tolerance in rice [16]; and the *AemNAC2* gene can reduce cadmium content in transgenic wheat grains [17]. However, most studies on NAC response to heavy metals have focused on model species and crops, and few studies have been conducted on woody plants.

*Salix integra* (*S. integra*) is a short-rotation woody plant in the *Salix* genus of the Salicaceae that is characterized by rapid growth, abundant tillers, and easy asexual reproduction. It can be used in weaving and is also an important bioenergy tree. In recent years, it has been found that it has a high tolerance to a variety of heavy metals [18,19], especially in the absorption of lead (Pb) up to approximately 20,000 mg/kg [20]. In addition, there is little upward transfer of Pb in *S. integra*, and the enrichment of Pb in the belowground tissues hardly affects the acquisition of clean biomass in the aboveground part, making it an ideal species for phytoremediation in Pb-contaminated areas. However, the identification of and research on the important Pb resistance transcription factor family in *S. integra* have not been reported. Our research group has found that some transcripts of the *S. integra* NAC, AP2/ERF, and WRKY families are induced under Pb stress, among which the NAC family has the most induced transcripts [21]. Therefore, identification of *S. integra* NAC (SiNAC) family members and analysis of their expression under Pb stress are prerequisites for mining important Pb-resistant NAC genes in *S. integra*.

In recent years, transcriptome sequencing has been widely recognized as an effective method for the identification of plant transcription factor families under specific conditions for species such as *S. integra*, whose genome data have not yet been published. For example, transcriptome-wide identification and expression analysis of the MYB, ERKY, and CCCH families of *Pinus massoniana* under abiotic stress, such as phosphorus deficiency [22,23,24], the WRKY family of *Eucalyptus globulus* under cold acclimation [25], and the R2R3-MYB family of barley under boron stress [26] have been completed. Full-length transcriptome sequencing (also known as third-generation sequencing, or NGS) technology based on the PacBio platform has been developed in recent years to provide longer read lengths and higher accuracy [27] than next-generation sequencing (NGS) technology. By combining it with NGS to identify transcription factor families, many highly accurate sequences for all expressed genes can be efficiently obtained. This method has been applied to the identification of the ERF family of *Actinidia valvata* under waterlogging stress [28] and the identification of the WRKY family related to *valsa* canker disease in *Malus sieversii* [29].

In this study, the transcriptome sequencing data for *S. integra* under Pb stress were used to identify and analyze the physicochemical properties of Pb-responsive SiNAC. Phylogenetic analysis, motif prediction, and temporal expression profile analysis of Pb stress were performed to identify the candidate Pb response *SiNAC*. Bioinformatics analysis was used to predict the target pathway, which was the basis for subsequent functional verification of important *SiNAC* genes and provided valuable references for the mining of heavy metal tolerance genes in other woody plants.

## 2. Results

### 2.1. Identification of Pb Response SiNAC Transcription Factors

A total of 55 eligible candidate NAC proteins were identified from the *S. integra* transcriptome data under Pb stress using HMM files of the NAC domain. A total of 504 similar proteins were obtained by homologous comparison between the NAC sequences of *P. trichocarpa* and the transcriptome data of *S. integra*. After taking the intersection of two data sets, 55 common sequences are obtained. After Pfam and SMART identification, all 55 sequences contained NAC domains. After manual deletion of two excessively short sequences, a total of 53 NAC transcripts of *S. integra* were obtained. According to transcriptome sequencing data, the amino acid lengths of 53 SiNAC proteins were calculated, as shown in Table 1. Most of the SiNAC proteins were between 200 and 600 aa in length; only 2 SiNACs were less than 200 aa, and 7 SiNACs were longer than 600 aa.

The subcellular localization and physicochemical property information, such as MW, PI, and GRAVY, of 53 SiNAC proteins predicted through the website are shown in Table 1. The MW value was between 17,000 and 76,000, the PI was up to 9.71, indicating that the molecular structure was stable, and all 53 SiNACs were hydrophilic proteins. In addition, the protein sequences of 53 SiNACs based on full-length transcriptome sequencing data are listed in Table S1. The prediction results of subcellular localization showed that 30 SiNACs had nuclear localization, and no localization information was detected for the rest.

### 2.2. Classification and Naming of SiNACs

To study the taxonomic and evolutionary relationships of SiNACs, we used MEGA 11 to perform sequence alignment between *S. integra*, *A. thaliana,* and *P. trichocarpa* and then constructed a phylogenetic tree. Arabidopsis thaliana is a model plant, and its NAC protein family members are well classified, which can help to determine the subfamily classification of SiNACs. Poplar is a model species for woody plants such as *S. integra* and belongs to the Salicaceae family, with the two species being closely related to each other, which can provide a reference for the naming of SiNACs.

**Figure 1:**
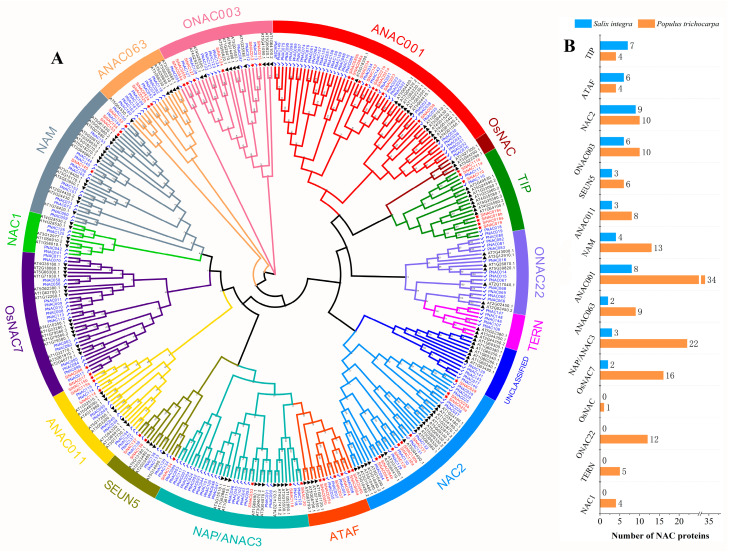
The phylogenetic trees of 53 SiNACs of *S. integra*, 163 PNACs of *P. trichocarpa,* and 134 AtNACs of *A. thaliana* were constructed using the NJ method of MEGA 11 software (**A**), and the number of proteins encoded by Pb-response *SiNAC* transcripts and the number of PNACs identified in the genome-wide *P. trichocarpa* of each subfamily were counted (**B**). The red stars represent SiNAC, blue checks represent PNAC, black triangle represent AtNAC.

After sequence alignment of 134 AtNAC proteins, 163 PNAC proteins, and 53 SiNAC proteins, the constructed phylogenetic tree was generated, as shown in Figure 1A. In the phylogenetic tree, all SiNACs were more closely related to PNACs than to AtNACs, so we named 53 SiNACs according to the corresponding PNACs. Of the 350 NAC proteins, except for 7 AtNACs and 5 PNACs that were not classified, all other sequences were grouped into 15 subfamilies. SiNACs were only distributed across 11 subfamilies and were not in the NAC1, TERN, ONAC22, or OsNAC subfamilies. We statistically analyzed and compared the NAC numbers of all the identified subfamilies of *S. integra* and *P. trichocarpa* (Figure 1B) and found that the number of *SiNACs* expressed in different subfamilies did not correspond to that of *P. trichocarpa*. In the TIP and ATAF subfamilies, the number of proteins encoded by *S. integra* Pb-responsive NAC transcripts exceeded that identified in *P. trichocarpa* genome-wide. In the NAC2 subfamily, the number of proteins encoded by the Pb-responsive transcripts of *S. integra* was also close to that of the members of *P. trichocarpa*, while some subfamilies had very few Pb-responsive transcripts (e.g., OsNAC7). The NAC2 subfamily is also the most abundant subfamily of NAC proteins, with 9, followed by ANAC001 and TIP.

### 2.3. Transcript Structure Analysis of SiNACs and Domain and Motif Prediction of SiNACs

**Figure 2:**
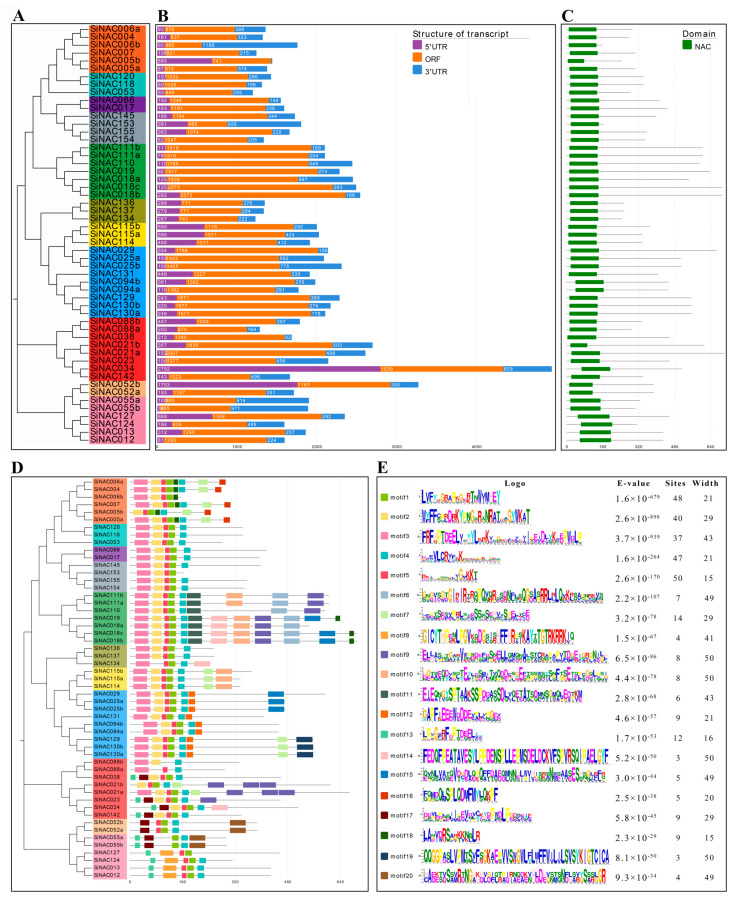
Phylogenetic tree construction (**A**), transcript structure analysis (**B**), NAC domain location prediction (**C**), motif prediction (**D**) of 53 SiNACs, and amino acid sequence and information of 20 motifs (**E**). The NAC domain and its location were detected by Pfam. MEME was used to predict motifs. Different colors represent different motifs.

To further explore the properties of *S. integra* NAC family proteins, only 53 SiNAC proteins were used to construct a phylogenetic tree by using the same method, and it was found that the classification results were consistent with those in 3.2 (Figure 2A). Based on transcriptome sequencing data, we calculated the 5′ UTR, ORF, and 3′ UTR lengths of transcripts encoding all identified SiNACs and visualized them using the tools in EvolView (Figure 2B). The lengths of the *SiNAC034* and *SiNAC052b* transcripts were extremely long, mainly because their 5′ UTRs were significantly longer than those of the other genes. The transcript length of the SEUN5 subfamily was the shortest. In addition, the Pfam website was used to predict the location of the NAC domain (Figure 2C). All the NAC domains were located at the N-terminus of the protein. Except for four SiNACs with shorter domains, the location and length of the domains of the other SiNACs varied little among the different SiNACs. The NAC domain lengths of SiNAC052a and SiNAC052b, the only two proteins in the ANAC063 subfamily, were similar and slightly shorter than those of the other 49 SiNACs. The NAC domain lengths of SiNAC005b and SiNAC021b were significantly shorter than those of other SiNACs.

Previous studies have shown that proteins belonging to the same group always have similar conserved motifs [30], which determine the similarity of protein structures. The results for the conserved motif analysis of all SiNACs are shown in Figure 2D. SiNACs belonging to the same subfamily often have similar motifs and motif arrangements. The number of motifs was set to 20, and the amino acid sequences of each motif are shown in Figure 2E. Motif1, motif4, and motif5 were identified in most SiNACs. Motif 1 was not present in SiNAC088a and SiNAC088b; motif 4 was not present in SiNAC006b, SiNAC153, and SiNAC127; and motif 5 was not present in SiNAC088b, SiNAC055a, and SiNAC055b. At the subfamily level, motif 2 and motif 3 were found in all subfamilies except ANAC063 and ONAC003. In addition, some motifs existed only in specific subfamilies, such as motif 16 being only in the ATAF subfamily, motifs 6 and 11 being only in the TIP subfamily, and motifs 12 and 19 being only in the NAC2 subfamily. TIP subfamily SiNACs had the largest number of motifs, with most containing 9–12. In addition, SiNAC021a of the ANAC001 subfamily also had 9 motifs, but SiNAC088a and SiNAC088b, which contain the fewest motifs, also belong to this subfamily, with only 3 motifs.

### 2.4. Temporal Expression Profile and STEM Analysis of SiNACs under Pb Stress

**Figure 3:**
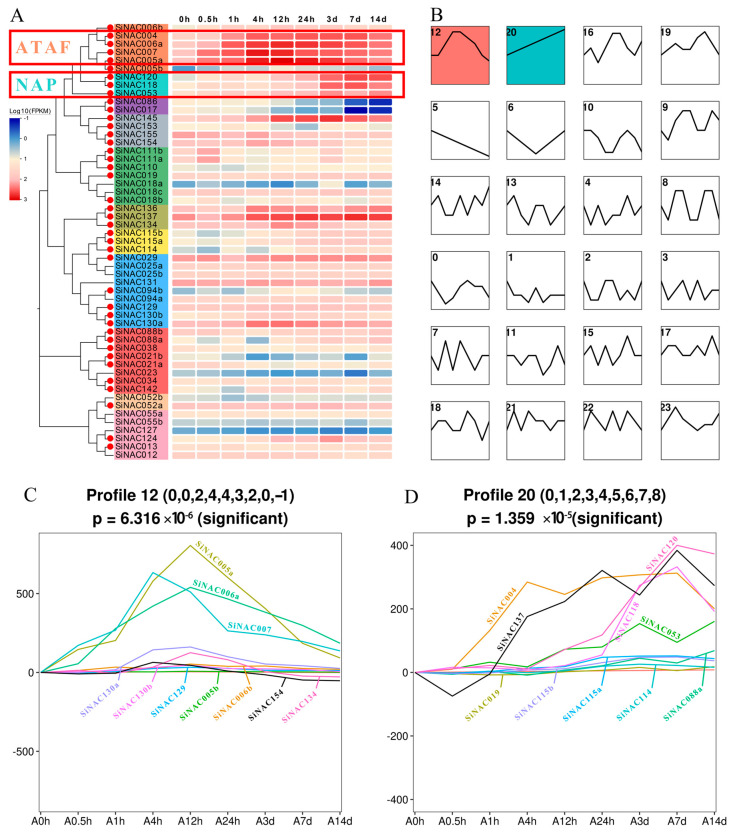
Pb response analysis of *SiNAC* transcripts. (**A**), Expression profiles of 53 *SiNACs* in *S. integra* roots at 9 time points (0 h, 0.5 h, 1 h, 4 h, 12 h, 24 h, 3 d, 7 d, 14 d) under Pb stress. After adding 1 × 10^−6^ to the FPKM value, log10 was standardized and centralized. Blue represents low expression levels, and red represents high expression levels. Evolview was used to draw a heatmap. (**B**), STEM analysis of Pb response patterns in 53 *SiNACs*. A white background color for a profile indicates no significant change pattern, while other colors indicate a significant change pattern. (**C**), The FPKM values of 10 *SiNAC* transcripts included in Profile 12 under Pb stress. (**D**), The FPKM values of 10 *SiNAC* transcripts included in Profile 20 under Pb stress.

To reveal the role of NAC in response to Pb stress, the FPKM values of *SiNAC* transcripts encoding 53 *S. integra* NAC proteins under Pb stress were statistically analyzed based on RNA-seq data and presented in a heatmap (Figure 3A). As shown in the figure, the response of *SiNACs* to Pb stress was similar in some subfamilies and different among different subfamilies. The ATAF, NAP, OsNAC7, and SEUN5 subfamilies showed consistent responses to Pb stress. Among the four subfamilies, only OsNAC7 showed a negative response, while the others showed a positive response. There was no consistent pattern in the responses of the other seven subfamilies to Pb stress. Combined with previous identification results of differentially expressed transcripts (DETs, *p* < 0.05) [21], we found that 41 out of 53 *SiNACs* (marked by red dots in Figure 3A) were DETs, accounting for 77.3%. These results indicate that these *SiNACs* had a significant response to Pb stress at at least one time point. All transcripts of the ATAF, NAP, OsNAC7, NAM, SEUN5, and ANAC011 subfamilies showed significant Pb responses. The TIP, NAC2, ONAC003, and ANAC063 subfamilies showed significant Pb responses only in some of the transcripts, and the remaining 12 *SiNACs* showed no significant differential expression under Pb stress.

Moreover, STEM software was used to classify the Pb response patterns of 41 *SiNACs* according to FPKM values. *SiNACs* with the same Pb response patterns were classified into a profile, and the results are shown in Figure 3B. Among the 24 profiles, only Profile 12 and Profile 20 had a significant (*p* < 0.05) Pb response trend, while the other profiles had no significant Pb response trend. Profile 12 was the most significant (6.316 × 10^−6^) and contained 10 *SiNAC* transcripts. The trend of the Pb response first increased and then decreased, and the expression level peaked at approximately 12 h, showing a positive response in the early stage (within 24 h) of Pb stress. Among the three genes with the most obvious response were *SiNAC005a*, *SiNAC006a*, and *SiNAC007*, all belonging to the ATAF subfamily (Figure 3C). The significance of Profile 20 was 1.359 × 10^−5^. This profile also contained 10 *SiNAC* transcripts, and its expression level showed a trend of continuous increase. Five transcripts were the most obvious Pb-responsive *SiNACs*, including all transcripts *SiNAC120*, *SiNAC118,* and *SiNAC053* from the NAP subfamily, *SiNAC004* from the ATAF subfamily, and *SiNAC137* from the SEUN5 subfamily. All *SiNACs* of the NAP subfamily showed a positive response at the late stage (after 3 d) of Pb stress (Figure 3D). Therefore, based on the degree of Pb induction of transcripts, we suggest that the ATAF and NAP subfamilies are two important Pb response subfamilies with different Pb response patterns. Finally, *SiNAC004*, *SiNAC005a*, *SiNAC006a*, and *SiNAC007* of the ATAF subfamily and all three transcripts, *SiNAC120*, *SiNAC118,* and *SiNAC053* of the NAP subfamily, which were strongly induced by Pb, were selected as candidate lead-responsive *SiNAC* transcripts for further analysis (red box in Figure 3A).

### 2.5. Protein Sequence Analysis and Structure Prediction of 7 Candidate SiNACs

**Figure 4:**
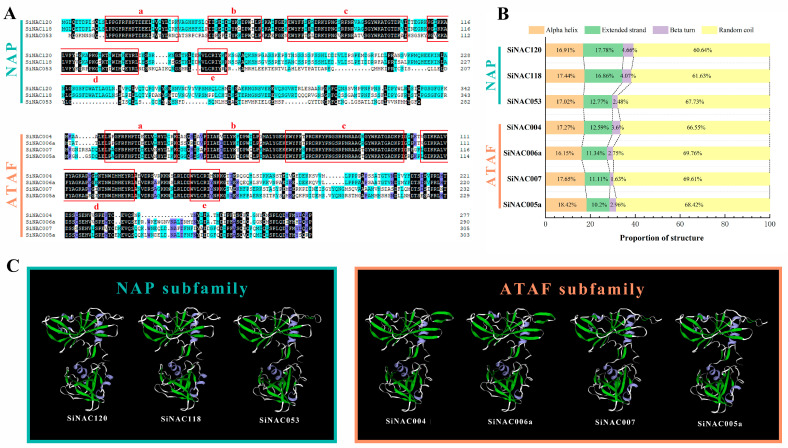
Amino acid sequence alignment (**A**), secondary (**B**), and tertiary (**C**) structure analysis of 7 candidate Pb response SiNACs. a-e represent the five subdomains (indicated by the red box in the figure) of the NAC domain. SOPMA and Swiss-model were used to predict secondary and tertiary structures.

To further analyze the seven candidate SiNACs, we compared the amino acid sequences of candidate SiNACs from two subfamilies separately. It was found that all SiNACs were conserved in the first 150 aa of the N-terminal, while the C-terminal had abundant variation. In particular, the sequence difference between the two subfamilies was greater. In the NAP subfamily, the sequences of SiNAC120 and SiNAC118 were more similar. In the ATAF subfamily, the SiNAC004 and SiNAC006a sequences were more similar, and the SiNAC007 and SiNAC005a sequences were more similar (Figure 4A). The results for the prediction of the secondary structure of seven SiNACs are shown in Figure 4B. The seven proteins were composed of an alpha helix, an extended strand, a beta turn, and random coil secondary structures. Among them, the proportion of random coils in seven proteins was more than 60%, even in SiNAC006a, which accounted for 69.76%. Beta turn accounted for the least among the four structures, less than 5%, and only 1.63% in SiNAC007. The proportion of the extended strand of SiNAC120 and SiNAC118 was higher than that of other SiNACs. The proportion of alpha helices showed little change among the seven SiNACs, ranging from 16–19%. The tertiary structure prediction results of approximately 150 aa at the N-terminus of seven NAC proteins are shown in Figure 4C, which shows that these parts have similar structures, are highly conserved, and are all homologous dimers. Meanwhile, the tertiary structures of SiNAC120 and SiNAC118, SiNAC004 and SiNAC006a, and SiNAC007 and SiNAC005a are similar.

### 2.6. Functional Enrichment Analysis of Associated Genes of 7 Candidate SiNACs

**Figure 5:**
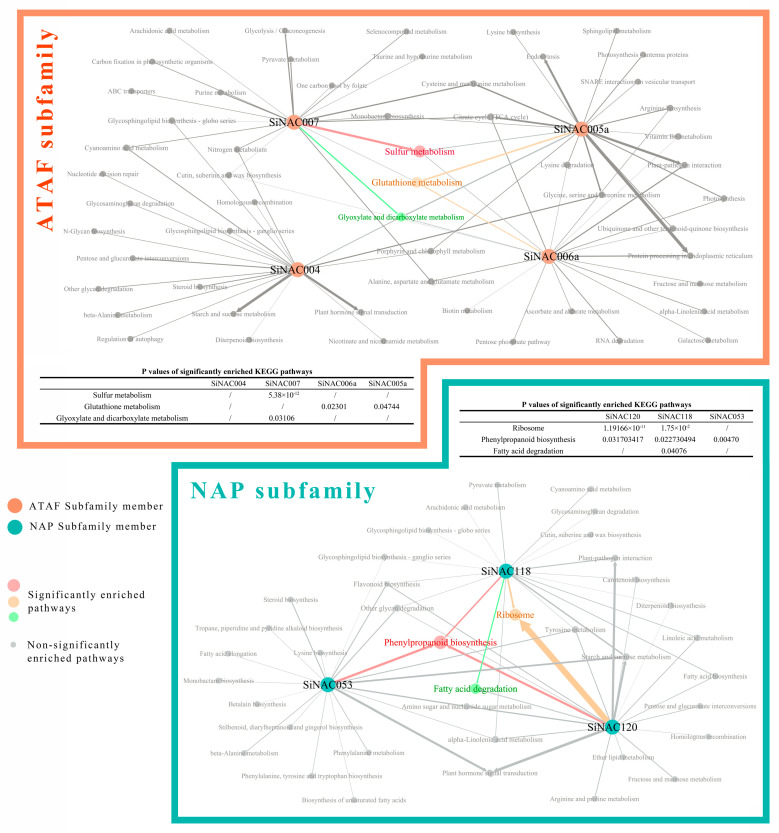
Target pathway prediction of 7 candidate *SiNACs* in the ATAF and NAP subfamilies. KEGG enrichment analysis was performed on four candidate transcripts of the ATAF subfamily and three candidate transcripts of the NAP subfamily using KOBAS 3.0. Gephi0.10.1 software was used to draw the figures.

To investigate the role of seven candidate SiNACs in Pb resistance, we set uniform confidence levels for each subfamily (0.085 for ATAF and 0.16 for NAP). From the previous WGCNA results [21], the associated genes of each *SiNAC* transcript were screened. KEGG enrichment analysis was performed on the associated genes of each SiNAC, and *p* < 0.05 was considered significant enrichment, as shown in Figure 5. In the ATAF subfamily, the associated genes of *SiNAC007* were significantly enriched in the “sulfur metabolism” and “glyoxylate and dicarboxylate metabolism” pathways, and the number of significantly enriched associated genes in the “sulfur metabolism” pathway was the largest. Both *SiNAC006a* and *SiNAC005a* were significantly enriched only in the “glutathione metabolism” pathway, and *SiNAC005a* enriched more associated genes in this pathway than *SiNAC006a*, but the enrichment in other pathways was not significant. In the NAP subfamily, the associated genes of the three *SiNACs* were significantly enriched in the “phenylpropanoid biosynthesis” pathway, which was the most enriched pathway. The associated genes of *SiNAC053* were only enriched in this pathway, while many associated genes of *SiNAC120* were significantly enriched in the “ribosome” pathway, and a small number of associated genes of *SiNAC118* were significantly enriched in the “ribosome” and “fatty acid degradation” pathways.

### 2.7. Subcellular Localization of SiNAC004 and SiNAC120

**Figure 6:**
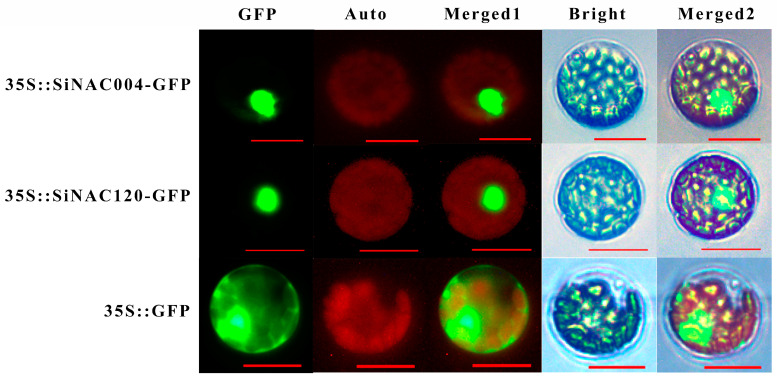
Subcellular localization of SiNAC004 and SiNAC120 in poplar mesophyll cell protoplasts. The 35 S::GFP fusion protein was used as a positive control and was detected throughout the nucleus and cytoplasm in poplar. Auto, chloroplast autofluorescence; Merged 1, merged GFP, and Auto images; Merged 2, merged bright, and Merged 1 images. The scale bar represents 10 μm.

To verify the subcellular localization of SiNACs, we randomly selected a SiNAC from the ATAF and NAP subfamilies for subcellular localization experiments. The subcellular localization results for SiNAC004 and SiNAC120 are shown in Figure 6. Both SiNAC004 and SiNAC120 are nuclear localization proteins.

### 2.8. Expression Verification of Seven Candidate SiNACs under Pb Stress

**Figure 7:**
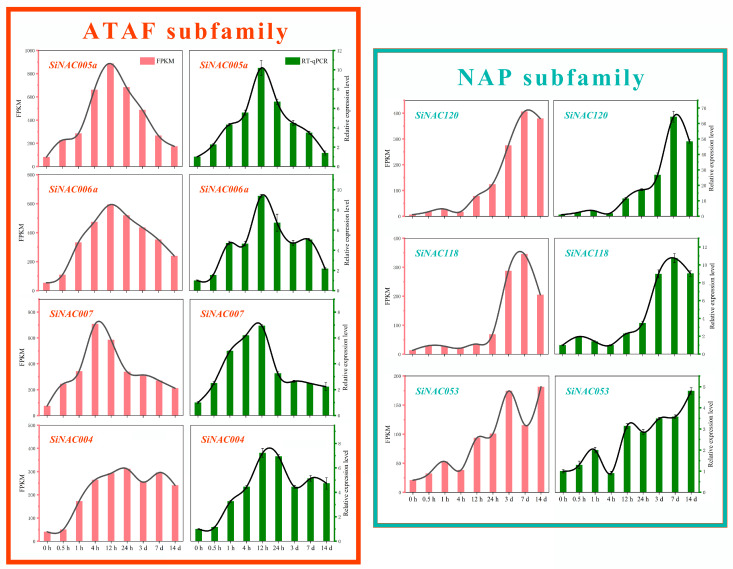
Relative expression levels of four (SiNAC005a, SiNAC006a, SiNAC007, and SiNAC004) and three (SiNAC120, SiNAC118, and SiNAC053) candidate Pb-responsive transcripts of the ATAF and NAP subfamilies, respectively, identified via RT–qPCR at nine time points under Pb stress. Draw with Origin 2018.

To further determine the response of candidate *SiNACs* to Pb stress, RT-qPCR was used to detect the relative expression levels of 7 transcripts under corresponding Pb stress times, and the results are shown in Figure 7. First, it can be seen that the changing trend of the relative expression level of all transcripts is basically consistent with the changing trend of the FPKM value obtained by sequencing. Second, we were able to confirm that transcripts from different subfamilies have different response patterns to Pb. The Pb response of ATAF subfamily transcripts peaked at 4–24 h and fell back at 3–7 d, while the Pb response of NAP subfamily transcripts peaked at 3–7 d and increased slowly at 4–24 h. The expression peaks of ATAF subfamily transcripts were generally higher than those of NAP subfamily transcripts.

## 3. Discussion

NAC is an important transcription factor related to abiotic stress tolerance in plants. In recent years, NAC has also been found to play an important role in plant tolerance to heavy metals such as cadmium, zinc, and copper [18,19]. As a short-rotation woody plant, *S. integra* has great phytoremediation potential for Pb and other heavy metals [20]. However, there has been no study on the Pb tolerance of NAC transcription factors in woody plants. Transcriptome-wide familial identification can use transcriptome data under various stresses to identify genes that specifically respond to the stress. Bail et al. [28] and Aguayo et al. [25] identified 131 waterlogging response *AvERF* genes (transcripts) and 51 cold acclimation response *EglWRKY* genes (transcripts) in *Actinidia valvata* and *Eucalyptus globulus*, respectively. In this study, 53 Pb-responsive *SiNAC* transcripts were identified by analyzing *S. integra* transcriptome data under different Pb stress times. A total of 41 out of 53 *SiNACs* had significant Pb responses. After STEM analysis of 41 *SiNACs*, 4 and 3 candidates from the ATAF and NAP subfamilies with the most significant Pb responses were selected for further analysis.

### 3.1. The Number and Characteristics of Pb Response SiNAC Transcripts

In the absence of *S. integra* genomes, we identified 53 Pb-responsive *NAC* transcripts from *S. integra* Pb-resistant clones for the first time using a relatively cost-effective combination of NGS and TGS. As shown in Figure 1A, compared with *A. thaliana*, all SiNAC proteins are in the same branch as PNAC but are far away from ANAC, which is the result of the close relationship between poplar and willow. For this reason, the total NAC quantity of *S. integra* at the genome level may be more similar to that of poplar. A total of 163 NAC members were identified in *P. trichocarpa* genome wide [31], and 53 Pb-responsive *NAC* transcripts were identified in *S. integra*, suggesting that the NAC family plays an important role in *S. integra* tolerance to Pb.

From the comparative analysis of NAC numbers in each subfamily, it was found that the number of SiNAC encoded by the Pb-responsive transcripts of the TIP and ATAF subfamilies even exceeded the number of members of corresponding families identified in the genome-wide analysis of *P. trichocarpa* (Figure 1B). We compared the transcript sequences of all the proteins in the first three subfamilies where SiNAC is abundant. SiNAC018a, SiNAC018b, and SiNAC018c, among the seven SiNACs of the TIP subfamily, may be the proteins encoded by 3 different transcripts produced under Pb stress of the same gene, as well as SiNAC111a and SiNAC111b. In the ATAF subfamily, only SiNAC004 and SiNAC007 may be single transcripts, while the other two members, SiNAC005 and SiNAC006, both produced two transcripts under Pb stress. The nine transcripts of the NAC2 subfamily may also contain three pairs of different transcripts from the same gene and three single transcripts. We speculated that Pb stress might induce the transcription of *S. integra* genes to produce multiple different transcripts, and Figure 3A shows that they had different Pb response expression profiles. These results suggest that some subfamily genes may regulate the response of *S. integra* to Pb by producing multiple transcripts.

Transcriptome-wide identification of the heavy metal response NAC in other species has not been reported. Wang et al. [32] identified 21 NACs from *Tamarix hispida* transcriptome data at 4 time points under NaHCO_3_ stress. The results indicated that the NAC quantity of *Tamarix hispida* under saline-alkaline stress induced by external application of NaHCO_3_ was much less than that of *S. integra* under Pb stress. This suggests that the response of the NAC family to Pb stress is extensive in *S. integra*. This may also be because this study used transcriptome data from nine (9) time-point samples.

### 3.2. Motif and Pb Response Characteristics of SiNAC of the ATAF and NAP Subfamilies

Consistent with the highly conserved structural features of the N-terminus of NAC transcription factors, the most conserved motifs 1, 4, and 5 in this study were located on the NAC domains of the N-terminus of almost all SiNACs (Figure 2D). Similar patterns were also found in NAC motif studies on woody plants such as *Camellia sinensis* (L.) O. Kuntze [33] and *Jatropha curcas* L. [34]. However, the highly distinctive C-terminal motif only has similarities among members within subfamilies, which may lead to functional differences among different subfamilies. For example, among the two subfamilies with obvious positive Pb responses in this study, the ATAF subfamily generally had motif 7 and motif 16 at the C-terminus compared with the NAP subfamily, and motif 16 only existed in the ATAF subfamily (Figure 2D). Functionally, the expression levels of ATAF subfamily transcripts were significantly upregulated in the early stage of Pb stress (within 24 h), and NAP subfamily transcripts were significantly upregulated in the late stage of Pb stress (after 3 d) (Figure 3). This was also confirmed by the results of the RT-qPCR experiment to verify its expression level (Figure 7), which was also consistent with the Pb response pattern of *S. integra* in the phases we found [21]. However, no similar findings have been published in studies thus far. Whether differences in motifs between subfamilies will lead to differences in subfamily function remains to be further explored.

At present, studies on the resistance of ATAF and NAP subfamily members to heavy metals, especially Pb, have not been reported, and only some studies have confirmed their role in other abiotic stresses. Among them, there are relatively more studies on ATAF subfamily genes. Wan et al. [35] conducted RT-qPCR tests on *SmNAC19*, *SmNAC31,* and *SmNAC75*, three members of the ATAF subfamily of eggplant, and confirmed that they had significant responses to abiotic stresses such as low temperature, high temperature, drought, and hormones within 24 h. However, the peak value of the response to different stresses was different. Wang et al. [36] and He et al. [37] studied *CsATAF1* in cucumber and *GhATAF1* in cotton, respectively, and confirmed that ATAF family genes can improve plant tolerance to abiotic stresses such as drought and high salt. The involvement of NAP subfamily genes in abiotic stress tolerance has only been reported in model species, and *RD26* (AT4G27410) of the NAP subfamily of *A. thaliana* may be induced by drought and high salt stress [13]. However, the response of different subfamilies to abiotic stress at different times has not been reported.

### 3.3. Target Pathway Prediction Analysis of 7 Candidate SiNACs

Transcription factors usually regulate the expression of target genes positively or negatively by binding with cis-acting elements in the promoter region of specific target genes on DNA, thus affecting the function of target genes [38,39]. In this study, functional enrichment analysis was performed on the associated genes of seven candidate *SiNACs*. Target pathway prediction of ATAF subfamily transcripts revealed significant enrichment of the “glutathione metabolism” pathway, including the *glutathione S-transferase* (*GST*), *nicotinamide adenine dinucleotide phosphate* (*NADPH*), and *ascorbate peroxidase* (*APX*) genes, and its upstream “sulfur metabolism” pathway. The target pathway prediction results of NAP subfamily transcripts revealed significant enrichment of the “phenylpropanoid biosynthesis” pathway containing *peroxidase* (*POD*) and *β-glucanase* (*bgl*) genes. At present, research on the target genes of NAC transcription factors is relatively limited, and most studies have focused on model species. Fujita et al. [13] studied the stress-related gene *RD26* in the NAP subfamily of *A. thaliana* and found that the *GST* gene in the “glutathione metabolism” pathway was significantly upregulated in overexpressed plants. The *OsNAC6* gene from the ATAF subfamily was found to be associated with drought and high salt tolerance in rice by Nakashima et al. [40]. It was found that its target genes encode not only GST and NAD(P)H-dependent redox enzymes belonging to the “glutathione metabolism” pathway but also POD and bgl proteins belonging to the “phenylpropanoid biosynthesis” pathway. Fang et al. [41] also found that the *SNAC3* (Os01g09550) gene of the rice ONAC4 subfamily and its target genes (*OsAPX8*, *OsRbohF,* and *OsCATA*) were significantly coexpressed in both *SNAC3*-overexpressing and RNAi rice, and the tolerance of overexpressed plants to abiotic stresses such as high temperature and drought was significantly enhanced. The *APX* gene also belongs to the “glutathione metabolism” pathway. Therefore, the “glutathione metabolism” pathway may be regulated by multiple NAC subfamilies and is a widely regulated target pathway. Based on this, we can conduct subsequent gene function verification studies according to predicted target pathways and target genes.

Among the 53 *SiNACs* identified in this study, 41 showed a significant response to Pb stress. Not only the seven candidate transcripts of the ATAF and NAP subfamilies but also the *SiNAC137* transcripts of the SEUN5 subfamily showed a significant positive response to Pb stress and were significantly induced at 4 h to 14 d after Pb treatment. In addition, most SiNACs of the NAC2, NAM, and ANAC011 subfamilies also showed significant Pb responses. These transcripts may also play an important role in *S. integra’s* resistance to Pb stress. In *A. thaliana*, *ANAC004* from the ANAC001 subfamily positively regulates cadmium tolerance [14], while *ANAC017* from the NAC2 subfamily negatively regulates aluminium tolerance [15]. In this study, in addition to the positive response subfamilies, OsNAC7 and other subfamilies containing negative response transcripts may also participate in the Pb response of *S. integra* by reducing the activation of target genes. Paoli et al. found that the absorption of Pb and Cd ions in lichens would compete with the binding sites of Cu and Zn ions [42], which are also bivalent cations, affecting the absorption of micronutrients by lichens. The deficiency of Cu and Zn may inhibit the expression of related genes. However, Wang et al. [43] found that the OsNAC7 subfamily had both positive and negative responses to drought and salt stress in *Chrysanthemum nankingense*, which may be caused by differences in stress types and species. In addition, transcription factors are involved in plant life by regulating target genes. Even some transcription factor genes with low expression abundance may play an important role in the plant stress response. In this study, only seven candidate SiNACs in the two significant positive response patterns were further structurally analyzed, and target pathway prediction was carried out, while the structural and functional predictions of other SiNACs need further in-depth study. Furthermore, not only NAC transcription factors but also other transcription factor families may play a role in plant Pb tolerance. Gao et al. found that many transcription factor families, such as bZIP, ERF, and GARP, can respond to Pb stress in maize [44]. Zhang et al. verified the positive regulatory effects of several *bZIP* genes on Pb tolerance in maize [45], and Hou et al. also found that the *ZmbZIP54* gene could regulate Pb tolerance in maize by targeting the *ZmPRP1* gene [46]. The Pb resistance function of more transcription factors needs to be verified in the future.

## 4. Materials and Methods

### 4.1. Identification and Characterization of SiNAC Transcription Factors

The hidden Markov model (HMM) file of the NAC domain (PF02365) was downloaded from the Pfam protein family database (http://pfam.xfam.org/, accessed on 11 January 2022) and used for preliminary identification of the *S. integra* NAC protein in transcriptome sequencing data at different time points under the stress of 0.3 mM Pb(NO_3_)_2_ (approximately 99.4 mg/L) [21] by HMMER 3.0. The sequences of *Populus trichocarpa* (*P. trichocarpa*) NAC proteins were downloaded from the plant transcription factor database (PlantTFDB) (http://planttfdb.gao-lab.org, accessed on 8 December 2022). In order to identify the homologous proteins in *S. integra*, BLASTP was used to compare the *P. trichocarpa* NAC proteins to the *S. integra* NAC protein database (the e-value was set at 1 × 10^−5^). The reliability of the above two methods was set to 1 × 10^−5^, and the intersection of the screened sequences was taken. Pfam and SMART (http://smart.embl-heidelberg.de/smart/batch.pl, accessed on 20 January 2022) were used for further confirmation. After manually deleting sequences that were too short, the SiNAC proteins in response to Pb stress were determined. The Expasy website (https://web.expasy.org/protparam/, accessed on 1 July 2020) was used to predict the physical and chemical properties of the protein, such as molecular weight (MW), isoelectric point (PI), and grand average of hydropathicity (GRAVY). Subcellular localization of proteins was predicted using the ProtComp 9.0 tool available on the Softberry website (http://linux1.softberry.com, accessed on 25 October 2016). SOPMA (https://npsa-prabi.ibcp.fr/cgi-bin/npsa_automat.pl?page=npsa_sopma.html, accessed on 15 March 2021) and Swiss-model (https://swissmodel.expasy.org/, accessed on 29 July 2022) were used to predict the secondary and tertiary structures of proteins online. KEGG enrichment analyses were performed using KOBAS 3.0 [47].

### 4.2. Phylogenetic Tree Construction

The 134 AtNAC protein sequences of *A. thaliana* were obtained from PlantTFDB. The 163 PNAC protein sequences of *P. trichocarpa* from the research of Hu et al. [31] were downloaded. The sequences of the NAC protein of *S. integra* were compared with those of *A. thaliana* and *P. trichocarpa* using MEGA 11 software. The unrooted phylogenetic tree was constructed using the neighbor-joining (NJ) method available on MEGA 11 with 1000 bootstrap replicates. Beautify the phylogenetic tree with the tools available on the Evolview website (http://www.evolgenius.info/evolview/, accessed on 29 July 2022).

### 4.3. Transcript Structure Analysis and Domain and Motif Prediction

Based on the *S. integra* transcriptome sequencing results, information on the transcript encoding the SiNAC protein was obtained, including the length of the 5′UTR, ORF, and 3′UTR. The location information of the NAC domain of the identified SiNAC was predicted by the Pfam website. Evolview was used to visualize the results of the abovementioned analysis. The MEME online tool (https://meme-suite.org/meme/, accessed on 20 March 2022) was used to perform motif prediction on the SiNAC protein. The number of motifs was set to 20, and the maximum length was not more than 50.

### 4.4. Temporal Expression Profile and STEM Analysis of SiNACs under Pb Stress

Based on the transcriptome data of *S. integra* roots under Pb stress [21], the fragments per kilobase of transcript sequence per million base pairs sequenced (FPKM) value was used to characterize the expression level of *SiNAC* under Pb treatment at different time points. After adding 1 × 10^−6^ to the data, normalized log10 processing and centralized processing were carried out [48]. The time expression profile was generated in the form of a heatmap through the Evolview website. According to the Pb response expression profiles of all differentially expressed SiNAC transcripts, a series test of clusters was performed using short-time-series expression miner (STEM) [49] software version 1.3.13.

### 4.5. Subcellular Localization and Expression Verification of Candidate SiNACs under Pb Stress

#### 4.5.1. Plant Culture and Pb Treatment

The clone used in this study was obtained from the willow germplasm resource repository of the Jiangsu Academy of Forestry (Nanjing, China). The clone was selected by previous experiments and had a high tolerance to Pb [21]. The cuttings with a length of 15 cm and a diameter of 1 cm from an annual shoot of five-year-old willow were cultured hydroponically in inflatable planting baskets with a capacity of 10 L. After 2 weeks of culture, cuttings with consistent growth were selected and placed in a 1/4 Hoagland nutrient solution for another 2 weeks of culture. Pb(NO_3_)_2_ was then added to 1/4 Hoagland nutrient solution until the final concentration was 0.3 mM (approximately 99.4 mg/L). The roots were sampled at 0 h, 0.5 h, 1 h, 4 h, 12 h, 24 h, 3 d, 7 d, and 14 d of Pb treatment, frozen in liquid nitrogen, and stored in a −80 °C refrigerator for subsequent experiments. The aspen hybrid (*Populus davidiana* × *Populus bolleana*) clone Shanxin Yang used in the subcellular localization experiment was a long-term sterile tissue culture plant in our laboratory. The Murashige and Skoog (MS) medium (pH 5.8) contained 0.2% (*w*/*v*) Gelrite and 3.0% (*w*/*v*) sucrose. When the plants had grown for 35–45 days, large flat leaves were used for subcellular localization experiments. All of the seedlings were grown in a controlled environment growth chamber with LED lighting at 50 µmol·m^−2^s^−1^. The photoperiod was set at 16/8 h (light/dark), the temperature at 25/18 °C (day/night), and the humidity at 60–80%. Each sample contained three biological replicates.

#### 4.5.2. Subcellular Localization of SiNAC004 and SiNAC120

According to the sequence in the ORF region of the transcript, CE Design V1.04 was used to design specific primers containing enzyme restriction site sequences (Table 2). The ORF without a stop codon was cloned into the PJIT166-GFP vector by the ClonExpress II One Step Cloning Kit provided by Vazyme Biotech Co., Ltd. (Nanjing, China), and the 35S::SiNAC120/004-GFP fusion vector was constructed. The protoplasts of the clone Shanxin Yang were isolated using the method described by Tan et al. [50] and transformed using the PEG-mediated method. Green fluorescent protein (GFP) fluorescence was observed and recorded using a fluorescence microscope (Carl Zeiss, Oberkochen, Germany).

#### 4.5.3. Real-Time Quantitative PCR (RT-qPCR)

The SteadyPure plant RNA extraction kit provided by Accurate Biotechnology (Hunan) Co., Ltd. was used to extract total RNA from *S. integra* roots, and DNase I was used to remove genomic DNA. The extracted total RNA was then used for reverse transcription experiments using the *Evo M-MLV* RT premix for qPCR kit provided by Accurate Biotechnology Co. to synthesize *S. integra* cDNA.

Specific primers for candidate SiNAC transcripts (Table 2) were designed for RT-qPCR amplification, and SiActin1 (transcript4378) transcripts were used as standardized internal control genes [21]. RT-qPCR amplification was performed using SYBR green master mix on an ABI ViiA 7 Real-Time PCR system (Applied Biosystems, Carlsbad, CA, USA). The PCR procedure was as follows: 95 °C for 20 s; 45 cycles of 95 °C for 1 s and 60 °C for 20 s; and a melting curve stage consisting of 95 °C for 15 s, 60 °C for 1 min, and 95 °C for 15 s, which were used to detect the specificity of the PCR products. The expression levels of related transcripts were calculated using 2^−ΔΔCt^ [51]. Origin 2018 software was used for mapping.

## 5. Conclusions

The response of the NAC family to Pb is extensive and strong. A total of 53 Pb-responsive SiNACs were identified under Pb stress, and 41 of them had a significant Pb response. The 53 SiNACs belonged to 11 subfamilies, among which multiple subfamilies represented by TIP, ATAF, and NAC2 had a gene that produced more than 1 transcript under Pb stress, and different transcripts had different responses to Pb. Pb response STEM trend analysis revealed that 41 SiNACs had an unreported response characteristic, namely, there were two significantly different positive response modes (early and late), each containing 10 SiNACs. In the early Pb response mode, the expression peak was mainly reached at 24 h, and the three SiNACs with the highest response degree belonged to the ATAF subfamily. The late Pb response pattern was induced significantly after 3 d of stress, and the most responsive SiNACs were mainly from the NAP subfamily. However, the expression levels of all transcripts of the OsNAC7 subfamily were continuously downregulated under Pb stress at all times, showing a negative response. Four and three SiNACs from the ATAF and NAP subfamilies, respectively, with significant Pb response patterns were selected as candidate transcripts for further structural and functional analysis. The prediction of subcellular localization of the ATAF and NAP subfamilies and the experimental verification of SiNAC004 and SiNAC120, which were randomly selected from the ATAF and NAP subfamilies, respectively, indicated that they were nuclear localization proteins. Functional enrichment analysis of the associated genes of four SiNACs in the ATAF subfamily indicated that the target genes may belong to the “sulfur metabolism” and “glutathione metabolism” pathways. The target pathways of the three NAP subfamily SiNACs may be the “ribosome” and “phenylpropanoid biosynthesis” pathways.

## Figures and Tables

**Table 1:** Information on *SiNACs* and their predicted proteins.

Gene	Amino Acids Length (aa)	MW	PI	GRAVY	Subcellular Localization	Gene	Amino Acids Length (aa)	MW	PI	GRAVY	Subcellular Localization
*SiNAC052b*	388	42,952.59	4.95	−0.616	E (2.55)	*SiNAC055b*	294	33,002.79	7.07	−0.593	E (2.45)
*SiNAC018c*	690	75,707.17	4.65	−0.603	E (2.42)	*SiNAC052a*	388	42,952.59	4.95	−0.616	E (2.55)
*SiNAC021b*	611	68,713.77	5.09	−0.629	E (2.45)	*SiNAC094b*	453	50,832.32	5.71	−0.647	N (6.12)
*SiNAC021a*	668	75,133.32	5.13	−0.599	E (2.47)	*SiNAC131*	408	44,754.26	4.73	−0.391	N (6.18)
*SiNAC034*	512	56,842.01	4.76	−0.654	E (2.55)	*SiNAC094a*	453	50,832.32	5.71	−0.647	N (6.12)
*SiNAC018a*	545	60,263.18	4.75	−0.689	E (2.48)	*SiNAC038*	459	51,557.96	5.84	−0.557	E (2.26)
*SiNAC110*	594	66,132.69	5.25	−0.506	E (2.51)	*SiNAC114*	336	37,789.69	5.33	−0.734	N (8.82)
*SiNAC018b*	690	75,707.17	4.65	−0.603	E (2.42)	*SiNAC086*	415	46,912.09	6.06	−0.768	N (8.66)
*SiNAC029*	595	66,522.90	4.59	−0.475	E (2.38)	*SiNAC088a*	289	32,636.37	5.23	−0.780	E (2.66)
*SiNAC130b*	558	62,335.41	4.62	−0.585	N (7.27)	*SiNAC155*	357	40,265.41	7.61	−0.597	N (8.66)
*SiNAC019*	638	70,509.50	4.94	−0.495	E (2.42)	*SiNAC007*	306	34,952.25	5.50	−0.732	N (8.98)
*SiNAC127*	455	50,822.62	6.45	−0.828	E (2.72)	*SiNAC006a*	291	33,328.84	6.72	−0.660	N (9.04)
*SiNAC111b*	605	67,688.41	4.98	−0.564	E (2.39)	*SiNAC136*	256	28,925.80	9.15	−0.637	N (8.95)
*SiNAC025b*	474	54,195.17	4.71	−0.683	E (2.88)	*SiNAC134*	246	28,096.11	9.64	−0.647	N (8.57)
*SiNAC025a*	473	54,190.15	4.74	−0.692	E (2.93)	*SiNAC012*	429	47,885.04	4.87	−0.778	N (7.83)
*SiNAC129*	556	61,856.85	4.70	−0.547	N (7.32)	*SiNAC005b*	246	28,425.01	6.97	−0.852	N (8.74)
*SiNAC023*	458	51,635.31	5.36	−0.816	E (2.68)	*SiNAC124*	312	35,324.37	6.70	−0.863	N (7.16)
*SiNAC111a*	605	67,596.22	4.98	−0.585	E (2.35)	*SiNAC154*	348	39,172.56	8.72	−0.526	N (8.54)
*SiNAC115a*	336	37,996.11	5.42	−0.695	N (8.82)	*SiNAC053*	282	32,336.68	8.58	−0.683	N (9.00)
*SiNAC130a*	558	62,335.41	4.62	−0.585	N (7.27)	*SiNAC120*	343	38,290.14	8.56	−0.580	N (8.82)
*SiNAC153*	164	19,106.97	9.28	−0.794	N (8.95)	*SiNAC005a*	304	34,995.47	6.02	−0.776	N (8.68)
*SiNAC055a*	294	33,002.79	7.07	−0.593	E (2.45)	*SiNAC017*	396	44,977.08	6.07	−0.758	N (8.71)
*SiNAC013*	429	47,558.87	5.16	−0.740	N (7.33)	*SiNAC118*	344	38,270.96	8.46	−0.567	N (8.90)
*SiNAC145*	397	44,760.94	6.09	−0.666	N (8.69)	*SiNAC137*	256	28,963.74	9.04	−0.728	N (8.95)
*SiNAC088b*	333	37,767.38	5.94	−0.813	E (2.87)	*SiNAC004*	278	31,695.11	8.15	−0.583	N (8.97)
*SiNAC115b*	371	41,655.12	5.20	−0.668	N (8.80)	*SiNAC142*	340	38,530.56	5.52	−0.680	E (2.37)
*SiNAC006b*	154	17,813.56	9.71	−0.672	N (9.09)						

MW, molecular weight; PI, isoelectric point; GRAVY, grand average of hydropathicity; E, extracellular; N, nucleus.

**Table 2:** Primers used in this study.

Primer ID	Forward Primer (5′-3′)	Reverse Primer (5′-3′)
SiNAC120_166	tggagaggacagcccaagcttATGGGACTGCAAGAAACAGATCC	gctcaccatggatcctctagaCTGCTTAAACCCGAACCCACT
SiNAC004_166	tggagaggacagcccaagcttATGAAGGCGGCGGCATTA	gctcaccatggatcctctagaAAACGGCTTCTGCAAATACATGA
qSiNAC005a	GCTTCCAGAAATGGCACTGTATGGT	GGTTTATCCGCTCCGGTTGCT
qSiNAC006a	CGGCATGGCCTTGTATGGAGAAA	GCAGCACGATTCGGTCTCGAT
qSiNAC007	GGAGCGAAAGCCCGACATCA	TGGCATCACTCATCACCATCGC
qSiNAC004	TGTCGCATACACAACAAGAAAGGCA	GCACAGAATCTGACGTGTCGAAATACA
qSiNAC120	GATCCATGGCTCTTACCAAGCAAGG	GTTGGGTCGGGATCCATTCGG
qSiNAC118	GGTTGCCGGTCACCATTTCTCTT	GTTGGGTCGGGATCCATTGGG
qSiNAC053	AGAGCAACTGTGTCAGGGTATTGGA	TCGGTCTTGGTGCCCTTAGGT
SiActin1	AGGACCACGCCTTTGACAGC	CGAAAGGGAGTGGCGTGGAG

Uppercase letters represent gene sequences; Lowercase letters represent vector sequences.

## Data Availability

Not applicable.

## Supplementary Materials

The supporting information can be downloaded at: https://www.mdpi.com/article/10.3390/ijms241411334/s1.
